# Relationship between children’s skills in school subject learning and athletic ability

**DOI:** 10.3389/fpsyg.2022.1026563

**Published:** 2022-11-04

**Authors:** Syuro Ito

**Affiliations:** Faculty and Staff Personnel Department Guidance Officer, Sakai City Board of Education Secretariat, Sakai, Japan

**Keywords:** development, repeated side jump, softball throwing, subject learning, ability, dexterity

## Abstract

**Background and purpose:**

Japanese elementary school children are trained in arts and crafts, music, arithmetic, the Japanese language, life environment studies, physical education, and so on. Children must learn through doing as they develop physically, because the range of activities in their daily lives is still narrow. Subject learning is inseparable from daily life. Teachers should plan lessons with an awareness of the physicality of activities. Therefore, this study clarified the relationship between the ability for skillful and quick physical movement and subject learning among Japanese elementary school students.

**Methods:**

For the second to fourth grades of elementary school, the measurement results and subject evaluation (skill) were compared at the individual level. Students were divided into a group with high grades in subject learning and one with middle and lower grades, and a t-test was conducted to observe if there was a significant difference in the records of two physical activities: repeated side jump and softball throw.

**Main findings:**

Significant difference was found for repeated side jumps depending on whether the arts and crafts grades were high for all children in grades 2–4. Additionally, there was a significant difference in softball throwing records between the second and third graders, depending on whether the children’s performance in arts and crafts was high. Conversely, there was no significant difference between the second to fourth grade children’s musical scores and repeated side jump records. There was a negative correlation between sophomore and senior year music performances and the softball throwing record. Thus, the development of children’s dexterity is related to subject learning.

**Conclusion:**

Considering that elementary school teachers spend sufficient time with children on a daily basis teaching subjects such as physical education and music, it may be beneficial for them to be aware of dexterity’s importance across multiple subjects. Furthermore, it allows for an approach that engages children in physical activities from early childhood which can help in preparing them for learning a variety of subjects in school.

## Introduction

Elementary school children in Japan study subjects such as arts and crafts, music, arithmetic, Japanese language, life environment studies, and physical education. For children in the important transition period from infancy to childhood, learning in these subjects can serve as a basis for future learning, with a focus on cross-curricular and experiential learning at this stage (Ministry of Education, Culture, Sports, Science, and Technology, 2009). This is because children at this age are still trying to adapt to school-based education according to their developmental stage based on their natural behaviors in a narrow range of activities within daily life.

Thus, school education at this stage needs to be grounded in daily life and the developmental stages of children; this, in turn, also means that methods incorporating an awareness of physicality need to be used in lessons. At the elementary school stage, physical movement ability (dexterity) is an aspect of physicality required for a range of subjects. Additionally, childhood manual dexterity is gradually refined throughout development, with daily self-care (such as tying shoelaces), play (such as using a paintbrush), and academic tasks (such as handwriting), which are crucial (Gaul and Issartel, 2016; Fuelscher et al., 2021). For example, with regard to arts and crafts, children need appropriate skills for creating pictures and crafting, as well as skill and creativity for expressing themselves based on the characteristics of the available materials and tools. They also need to be able to use the equipment safely. For music, children need to be able to use their tongues and fingers correctly according to the type of instrument and to produce suitable dynamics. In arithmetic, appropriate motor skills include drawing (correct use of ruler); in Japanese language, students need to be able to hold a pencil correctly and maintain the appropriate pressure and directionality to create strokes when writing; further, when reading aloud and making presentations, students need to maintain an appropriate speaking style (i.e., loudness and speed). In physical education, the object of learning is the acquisition of physicality itself.

Studies have also suggested that manual dexterity has an impact on whether elementary school students enjoy learning using their fingers and learning that requires repetition and is related to engagement with diverse kinds of learning (Kawabata and Narumi, 2009, 2012) as well as students’ motivation to continue learning. Recently, however, manual dexterity has been declining due to factors such as changes in the way that children play, and initiatives to improve dexterity at the elementary school level are needed (Kawabata and Narumi, 2009, 2012).

Nonetheless, if teachers maintain an awareness of the physical development factors of individual children and provide instruction that is tailored accordingly, this may promote learning across subjects, rather than in just one subject, by instilling self-confidence in children, which can also have a positive effect on learning in later life.

The relationship between subject ability and dexterity has been studied from the perspective of home economics (Kawabata and Narumi, 2009; Narumi and Kawabata, 2013), as well as the aspect of the ability to perform calculations and manual dexterity in young children (Asakawa and Sugimura, 2009, 2011). Kawabata et al. demonstrated the relationship between learning to create objects and self-efficacy in the clothing field of home economics (Kawabata and Narumi, 2012) and also found that manual dexterity is related to a wider range of activities and learning (Kawabata and Narumi, 2009). An approach to subject learning (purpose) through the motor skill of handwriting (means) has also been attempted (Julius et al., 2016). Further, studies have shown that developing children’s physical fitness during elementary school age can enhance both motor and cognitive learning abilities related to academic performance (Abdelkarim et al., 2017). In addition, Gashaj and Trninić stated that “the existence of such a relationship is postulated in classical accounts of human development. In contemporary research, the existence of a relationship between motor development and the development of abstract concepts may form a crucial piece of evidence for theories of embodied cognition.” Existing research also suggests a link between fine motor skills in young children and various numerical and mathematical tasks (Gashaj and Trninić, 2022). Research is also being conducted on dexterity itself and on quantitatively and objectively measuring dexterity. However, even in these studies, the relationship between dexterity and athletic performance cannot be separated (Fuelscher et al., 2021; Makhija and Patel, 2022).

In this way, the development of children’s dexterity is an “old” and “new” problem, and although various approaches have been tried, this field of research is still developing, and a quantitative evaluation is also presenting difficulties. In addition, there is insufficient research on improving subject learning ability and dexterity from the viewpoint of whole body motor ability, centering on school physical education. This study aimed to clarify the relationship between physical development factors and the acquisition of abilities, mainly in the context of school subject learning. In terms of physical development factors, particular consideration will be given to physical movement ability (dexterity).

Terms relating to the ability to move correctly in time and space in physical activity: Attempting to move as much as possible in a short time: Agility Movement requiring accuracy in terms of time and space: Dexterity (Nagao and Okada, 2011). I believe that by clarifying the relationship between physical development factors and acquisition of subject learning ability, it is possible to provide appropriate teaching methods at an appropriate time for children, thereby improving their abilities and increasing their self-esteem.

## Material and methods

### Participants

The study participants were children in grades 2–4 at Elementary School A in Osaka Prefecture. The participants were 27 children in the second grade (27 in the repeated side jump and 23 in the softball throw [some children were absent from the latter]) (7–8 years old, *n* = 27, 14 boys (51.9%), 13 girls (48.1%)). Further, there were 33 students in the third grade (8–9 years old, *n* = 33, 17 boys (51.5%), 16 girls (48.5%)) and 31 students in the fourth grade (9–10 years old, *n* = 31, 15 boys (48.4%), 16 girls (51.6%). However, when there were events that students were unable to participate in, this reduced the number of participants. The number of participants is as shown in the table of results. The children’s performance in these tasks was measured in June 2011. In order to prevent subjective variation among evaluators in subject learning, one classroom teacher was required to evaluate all subject learning of the survey participants (approximately 23–33 individuals). In addition, the subjects of the study were a group of children in one class fixed from the beginning of the study to the end. The nervous system undergoes remarkable development in childhood, meaning that the earliest age from which accurate and objective learning evaluation can be performed to some extent is considered to be from the second grade. In addition, according to Skamon’s development curve, “development of the nervous system” (Scammon, 1930) is most prominent up to the age of 10 years (fourth grade of elementary school), so the participants in this study were from the second to the fourth grade of elementary school.

All participants were children enrolled in regular classes and did not require special individual support.

### Instruments

#### Use of the repeated side jump and softball throw tasks

The study focused on measuring children’s ability to perform repeated side jumps and softball throw, using these as a measure of children’s ability to move with skill and speed. The study also compares results for individual study participants with the evaluation (absolute evaluation) of skills for which children are required to move physically in subjects other than physical education (i.e., arts and crafts, music, arithmetic, and Japanese language). In other words, it was considered that children who scored well in items related to “skills” in subject learning would have good results in physical tasks that require dexterity, such as repeated side jump and softball throws.

The repeated side jump task measures agility and requires the ability to move skillfully while jumping repeatedly over a line of a fixed width within a fixed time.

The softball throwing task measures the ability to throw and to generate instantaneous force in the arms, shoulders, and chest. The ability to move with speed and skill is related to the height of the elbow in the pre-throwing posture and the timing with which the ball is released.

For each of the school subjects (arts and crafts, music, math, and Japanese), the teachers made final evaluations from A to C by summarizing the “skills” from September to December (A ranking the highest). The behaviors that are the subject of these “skill” metrics are whether children are able to use their bodies to perform skillful, accurate, or quick movements (may be related to repeated side jump and softball throwing skills). The items to be measured in the sports test were carefully selected from the viewpoint of whether they are related to “skill,” “accuracy,” and “speed.” The results in the repeated side jump and softball throw tasks were collected at school events (sports tests). Evaluations of subject learning were collected in daily school classes.

#### Evaluation of skills in arts and crafts among second-grade students

The same children who were evaluated in the sports tests described above were also evaluated in terms of “whether they were able to express themselves well” (i.e., their “skill”) in the subject of arts and crafts over the period from September to December. Children with an A grade were classified as the A group (higher-grade group), and others were classified as the B/C group (middle grade or lower).

The expressive ability (skill) in arts and crafts of the children in the groups was compared with individual children’s performance in the repeated side jump and softball throw tasks, making it possible to investigate the relationship between the results. Evaluations of the children’s expressive ability (skill) in arts and crafts were made based on whether they were able to meet the following three criteria:

Can the child express himself/herself imaginatively while also considering the beauty and usage of the results thereof?

Can the child express himself/herself through the use of color and shape?

Can the child use materials and tools according to their characteristics?

The evaluation criteria were based on the elementary school courses of study (Ministry of Education, Culture, Sports, Science, and Technology, 2009).

The evaluation was conducted over a short period from September to December. Expressive ability (skill) in arts and crafts probably contains a subjective emotional aspect. Thus, as a means of increasing reliability, the same teacher conducted evaluations from the same viewpoint (i.e., using the same evaluation criteria) during arts and crafts sessions. Below, the same applies to the subjects of music, arithmetic, and Japanese language. The specific evaluation criteria are as follows.

(1) Can the child use all of the paper when painting?

(2) Is there any ingenuity in the composition?

(3) Does the child devise colors until satisfied in response to expanding imaginative content?

(4) Does the child know how to use materials such as paints for various uses of color, markers, crayons, clay, and cardboard?

(5) Does the child use scissors and craft knives with an appropriate rhythm and while maintaining safety?

(6) When crafting, can the child craft objects that match what they want to express?

(7) Does the child pay sufficient attention to the details of their work?

(8) While this does not relate to expressive ability (skill), does the child have an attachment to their work in both painting and crafting?

The questionnaire uses the “teaching record,” which is a common index for evaluating the academic learning of Japanese elementary school students. Elementary school instruction manuals evaluate “knowledge/skills,” “motivation,” and “skills/expression” from A to C (Ministry of Education, Culture, Sports, Science, and Technology, 2009). Of these, A represents the highest grade. Additionally, in this study, we utilize the result of “skills/expression.” The teaching record (in this case, “research record”) makes use of a standardized table.

#### Evaluation of skills in music among second-grade students

To receive an A grade evaluation for expressive ability (skill) in music, students were assessed as to whether they met the following criteria:

Can the child play an instrument by paying attention to simple rhythms and tunes with an awareness of tone?

Absolute evaluation was used. The A group included the students who were evaluated most highly.

The objective of the elementary school subject of music is “to instill a love for music and sensitivity to music in students through activities of expression and musical appreciation and to cultivate basic musical abilities and musical taste” (Ministry of Education, Culture, Sports, Science, and Technology, 2009).

The specific evaluation criteria of skill were as follows:

(1) Can the child perform “Mushi no Koe” and “Yuyake Koyake” [Japanese children’s songs] faithfully to the score?

(2) The child’s tonguing with a recorder

(3) The strength of the child’s breath with recorder (sound strength)

(4) The child’s fingering of keyboard and recorder

#### Evaluation of arithmetic skills among second-grade students

Students were evaluated with regard to their skill in terms of dexterity in arithmetic based on their ability to draw figures, charts, diagrams, etc. In this context, there was a significant difference between students who received an A/B grade and those who received a C grade; therefore, both A and B were included in the higher-grade group (absolute evaluation).

The objective of the elementary school subject of arithmetic is “to use arithmetic activities to enable students to acquire foundational and basic knowledge and skills with regard to quantities and figures and to develop the ability to think and express themselves in a logical manner with a perspective on everyday events, while also enabling students to be aware of the fun of arithmetic activities and the positive aspects of mathematical processing, fostering an attitude of willingness to utilize them in daily life and learning” (Ministry of Education, Culture, Sports, Science, and Technology, 2009).

The specific evaluation criteria used here are as follows.

Does the child have a (conscious) understanding of the concept of a “right angle” aligned with ruled lines, and are they able to use a pencil to draw a right-angled triangle, square, or rectangle with a straight ruler or a triangle ruler?

#### Evaluation of skills in Japanese language

Students’ skill of dexterity in the Japanese language was evaluated based on whether they were able to write characters correctly. As with arithmetic, there was a significant difference between A/B grades and C grades; therefore, both A and B were included in the higher-grade group.

The objective of the elementary school subject of Japanese language is “to develop students’ ability to properly express and understand Japanese, improve their ability to communicate, and also foster ability in terms of thought, imagination, and a sense of language, as well as deepening their interest in Japanese, and developing an attitude of respect for Japanese” (Ministry of Education, Culture, Sports, Science, and Technology, 2009). The specific evaluation criteria are as follows:

(1) Can the child write carefully, paying attention to the shape of the characters, with the correct posture and grasp on the writing implement?

(2) Can the child write characters correctly according to the stroke order, paying attention to aspects such as the length and direction of strokes and how they touch and intersect?

(3) Can the child produce the tome, hane, and harai strokes when writing hiragana, katakana, and kanji (characters) that they already know as well as kanji that are new to them?

(4) Can the child hold a pencil correctly and write with stable pressure (regardless of whether they are left- or right-handed)?

Absolute evaluation was used.

#### Survey of students in the third and fourth grades of elementary school

The following four comparisons were made in the same way as for second-grade students. Relationship between the number of repeated side jumps and art (skill) evaluation. Comparison of high-grade group and middle- or lower-grade group by *t*-test. Relationship between the number of repeated side jumps and music (skill) evaluation. Comparison of high-grade group and middle- or lower-grade group by *t*-test. Relationship between the number of softball throws and art (skill) evaluation. Comparison of high-grade group and middle- or lower-grade group by *t*-test. Relationship between the number of softball throws and music (skill) evaluation. Comparison of high-grade group and middle- or lower-grade group by *t*-test. From the third grade onward, students perform on recorders in music class. Students use the keyboard harmonica until they reach the third grade, which requires both tonguing and fingering of the keyboard with the right hand. With the recorder, in addition to tonguing, students need to hold the recorder with their left hand and play using different and complex movements with the fingers of both hands. When playing the recorder, students also need to have a clear understanding of when to vary the strength of their breath to create dynamics, with the pieces that students play becoming more difficult in the third and fourth grades.

In arts and crafts, the complexity of crafting activities increases and students are expected to use chisels in the fourth grade. In painting, students increasingly use watercolor paints and are expected to paint still life in the third grade and portraits in the fourth grade. Watercolors require students to use several types of brushes and to be more precise in their movements (motor skills) when blending colors and painting.

### Data analysis

In this study, statistical analysis was performed using an unpaired *t*-test, as the population data were assumed to follow a normal distribution. The study sought to determine whether there is a relationship between children’s ability to perform repeated side jumps and softball throw and their ability to master learning content in terms of the skills included in the evaluation criteria for each subject. In other words, the study sought to determine whether participants who ranked highly in subject learning were able to perform a large number of repeated side jumps and how far they could throw a softball in a sports test. An unpaired t-test was performed to determine whether there is a relationship between these variables.

BellCurve for Excel Ver. 3.22 from Social Survey Research Information Co., Ltd., was used for statistical analysis.

Because this was a retrospective study, it was not possible to obtain participants’ consent. However, ethical considerations were fully discussed with the principal of Elementary School A at that time, and the study received approval on that basis. The data were also completely anonymized. The researchers have confirmed that there will be no problems with disclosing the content of the study to other researchers and educators.

This study was based on Ethics No. (HM-29) of the organization to which the researcher belongs. This study was conducted in accordance with the Declaration of Helsinki.

## Results

### Second-grade students’ results

Table 1 below shows the relationship between student achievements in the repeated side jump and their grades based on the evaluation of their skills in arts and crafts, music, arithmetic, and the Japanese language.

**TABLE 1:** Second-grade students’ skills in arts and crafts and other subjects and performance in the repeated side jump task (September–December 2011).

		Higher-grade group	Middle- and lower-grade group	*t*-value	Significance
Arts and crafts	Mean	30.7	26.6	2.5	**
	Variance	9.38	29.29		
	N	20	7		
Music	Mean	30.2	28.06	1.27	n.s.
	Variance	9.07	22.81		
	n	10	17		
Mathematics	Mean	29.63	27.73	1.14	n.s.
	Variance	12.78	26.02		
	n	16	11		
Japanese language	Mean	29.27	28.33	0.56	n.s.
	Variance	15.92	22.42		
	n	15	12		

**p < 0.05.

With regard to the repeated side jumps task, among second-grade students, a significant difference was observed at the 5% level between the group with higher skill grades and the group with lower skill grades in arts and crafts. However, it is unclear which one is the necessary condition and which is the sufficient condition. In addition, no significant difference was observed in any of the school subjects other than arts and crafts.

Table 2 below shows the relationship between student achievements in the softball throw tasks and their grades based on the evaluation of their skills in arts and crafts, music, arithmetic, and Japanese language.

**TABLE 2:** Second grade (AY 2011) arts and crafts and softball throwing task (September–December 2011).

		Higher-grade group	Middle- and lower-grade group	*t*-value	Significance
Arts and crafts	Mean	9.56	5.2	1.74	**
	Variance	29.32	4.2		
	n	18	5		
Music	Mean	6.11	10.21	–2.36	**
	Variance	4.36	35.57		
	n	9	14		
Mathematics	Mean	7.47	10.75	–1.49	n.s.
	Variance	15.98	44.21		
	n	15	8		
Japanese language	Mean	7.54	10	–1.14	n.s.
	Variance	14.27	42.67		
	n	13	10		

**p < 0.05.

A significant difference was observed at the 5% level between the group with higher skill grades and the group with lower skill grades in arts and crafts and in music. However, the necessary and sufficient conditions are unclear.

Further, the group with higher grades in music performed worse in the softball throw task.

**FIGURE 1:**
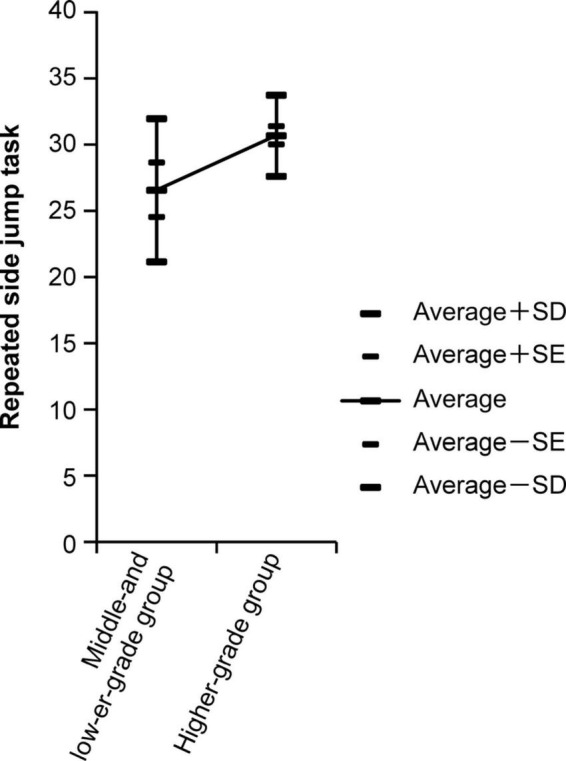
The number of repeated side jumps in the second grade and the results of ANOVA for the higher-grade group and middle- and lower-grade groups in arts and crafts.

Figure 1 shows the number of repeated side jumps in the second grade and the results of ANOVA for the higher-grade group and middle- and lower-grade groups in arts and crafts.

**FIGURE 2:**
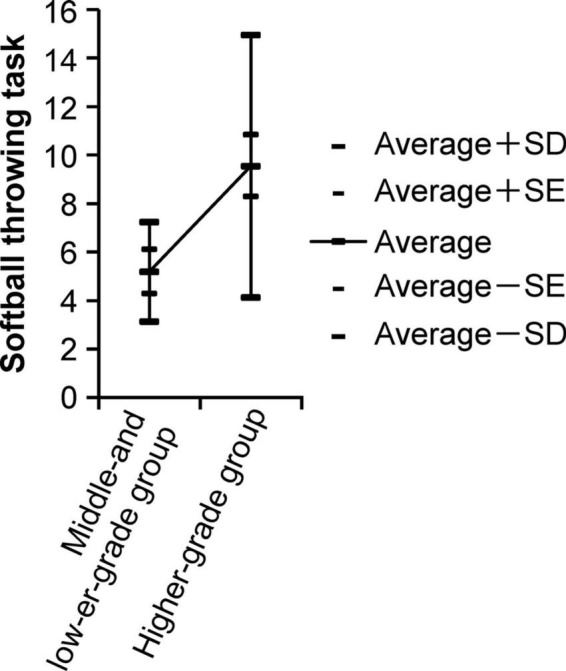
The distance of the second-grade softball throw and the results of the analysis of variance for the higher-grade group and middle- and lower-grade groups in arts and crafts.

In addition, Figure 2 shows the distance of the second-grade softball throw and the results of ANOVA for the higher-grade group and middle- and lower-grade groups in arts and crafts.

The results of the ANOVA reveal that the higher-grade group’s repeated side jumps and softball throws are better than the middle- and lower-grade groups.

### Third- and fourth-grade students’ results

Students in the third and fourth grades were tested in the same manner as those in the second grade, yielding the results presented in Tables 3, 4. In the third grade, a significant difference was observed at the 5% level between the high-ranking and low-ranking groups in terms of skills in arts and crafts (both repeated side jumps and softball throw). In the fourth grade, a significant difference was observed at the 5% level between the group with higher grades and the group with medium or lower grades in skills in arts and crafts (repeated side jump only).

**TABLE 3:** Third-grade students’ skill in arts and crafts and music and the repeated side jump task, softball throwing task (September–December 2011).

			Higher-grade group	Middle grade and lower group	*t*-value	Significance
Third-graders’ skill in arts and crafts and music and repeated side jump task	Arts and Crafts	Mean	31.64	27.63	1.95	**
		Variance	9.05	40.47		
		n	11	19		
	Music	Mean	29.20	32.50	–1.28	n.s.
		Variance	43.92	27.14		
		n	25	8		
Third-graders’ skill in arts and crafts and music and softball throwing task	Arts and Crafts	Mean	11.27	8.18	1.89	**
		Variance	39.16	9.76		
		n	22	11		
	Music	Mean	10.56	9.25	0.57	n.s.
		Variance	34.34	22.21		
		n	25	8		

**p < 0.05.

**TABLE 4:** Fourth-grade students’ skill in arts and crafts and music and the repeated side jump task, softball throwing task (September–December 2011).

			Higher-grade group	Middle- and lower-grade group	*t*-value	Significance
Fourth-graders’ skill in arts and crafts and music and the repeated side jump task	Arts and Crafts	Mean	37.28	33.47	2.45	**
		Variance	18.84	18.39		
		n	14	17		
	Music	Mean	36.00	34.80	0.74	n.s.
		Variance	8.49	29.33		
		n	11	20		
Fourth-graders’ skill in arts and crafts and music and the softball throwing task	Arts and Crafts	Mean	16.77	15.12	0.64	n.s.
		Variance	47.53	48.11		
		n	13	17		
	Music	Mean	13.27	17.32	–1.60	(Significant negative trend)
		Variance	23.82	56.01		
		n	11	19		

**p < 0.05.

**FIGURE 3:**
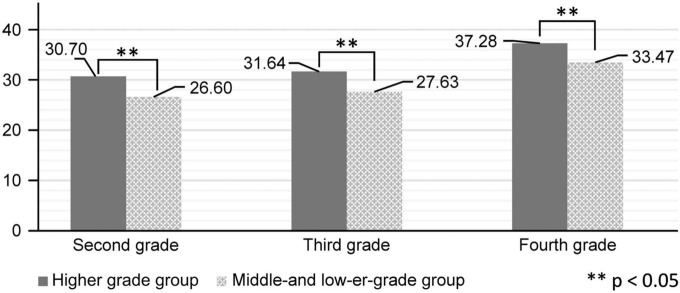
Arts and crafts and repeated side jump task.

**FIGURE 4:**
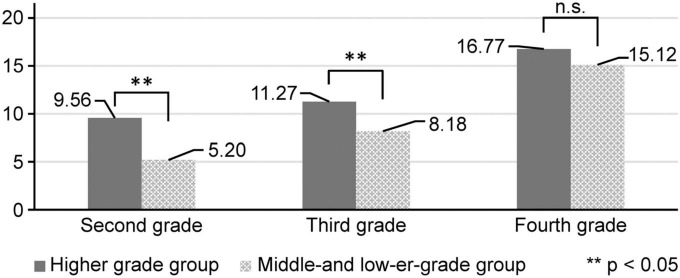
Arts and crafts and softball throwing task.

Figures 3, 4 show the changes in the results of drawing and the records of repeated side jumps, coupled with the results of drawing and the records of softball throws from the second grade to the fourth grade. Records are increasing for both higher-grade and middle- and lower-grade groups. In the repeated side jump, there is a significant difference in all three years; however, in the softball throw, there is no significant difference observed in the fourth grade.

### Summary of results

Table 5 shows the changes in the significant difference between arts and crafts and music in the repeated side jump by grade, and Table 6 shows the change in the significant difference in arts and crafts and music in the softball throw by grade.

**TABLE 5:** Differences in performance in arts and crafts and music, and changes in the significant difference in records for the repeated side jump by grade level.

		Second-grade	Third-grade	Fourth-grade
Arts and Crafts	*t*-value	2.50	1.95	2.45
	Significance	※※	※※	※※
Music	*t*-value	1.27	−1.28	0.27
	Significance	n.s.	n.s	n.s

**p < 0.05.

**TABLE 6:** Differences in performance in arts and crafts and music skills and changes in significant differences in softball throwing task records by grade.

		Second-grade	Third-grade	Fourth-grade
Arts and Crafts	*t*-value	1.74	1.89	0.64
	Significance	※※	※※	n.s.
Music	*t*-value	−2.36	0.57	−1.60
	Significance	Significant negative trend	n.s.	Significant negative trend

**p < 0.05.

As mentioned above, there was a significant difference in the number of repeated side jumps between the high-performing and non-high-performing groups in arts and crafts “skills” in all grades from the second to fourth grades. However, there was no significant difference in musical “skills” between the high-performing and non-high-performing groups in any grade (Table 5).

Conversely, there was a significant difference in the number of softball throwing tasks between the high-performing and non-high-performing groups in arts and crafts “skills” between the second and third-year students, but there was no significant difference in the fourth-year students. However, in terms of musical “skills,” there was a significant negative difference between the high-performing and non-high-performing groups between the second and fourth graders, and no significant difference was observed among the third graders (Table 6).

## Discussion

### Objectives of the study

This study aimed to clarify the relationship between physical development factors (physical movement ability (dexterity)) and the acquisition of abilities, mainly in the context of school subject learning.

### Main findings

This study’s findings showed a relationship between evaluations of students’ skills in the school subject of arts and crafts and the recorded number of repeated side jumps for students in the second, third, and fourth grades of elementary school. A relationship was also noted between evaluations of students’ skills in arts and crafts and the distance that they were able to throw a softball among students in the second and third grades. Students in the group with higher grades in arts and crafts were able to perform a higher number of repeated side jumps, and students in the group with middle or lower grades were able to perform a lower number of repeated side jumps. Conversely, there was no significant difference between the second to fourth grade children’s musical scores and repeated side jump records. There was a negative correlation between sophomore and senior year music performance and softball throwing record.

### Educational implications that these results entail

Macdonald et al. (2018) state that “significant positive associations were also apparent between academic performance and components of gross motor proficiency, specifically speed and agility, upper-limb coordination, and total gross motor scores.” Preliminary evidence from a small number of experimental studies suggests that motor skill interventions in primary school settings may have a positive impact on academic performance in mathematics and/or reading (Macdonald et al., 2018). This study also proves some of the results of previous studies.

In this study, the results of skills and repeated side jumps in the music department varied. In the second and fourth grades, there was a negative correlation between the results of softball throwing and musical skill, and this needs further investigation in the future. Many of the evaluation criteria used in this study depend on the cognitive and emotional factors that the subject emphasizes, and this was especially noticeable in music. As such, the function that controls the measured movements required for repeated side jumps and throwing a softball may differ from the function that controls the movements required in the subject of music. It may therefore be difficult to make a general comparison.

However, “rhythm,” a component of music, can be considered to be “dexterity” according to the criteria of “movement requiring accuracy in terms of time and space” (Nagao and Okada, 2011). Studies examining rhythmic movement and dexterity have been conducted (Asakawa and Sugimura, 2011), although little time was allocated in the present study to evaluate “rhythm” skills in music.

Prior studies have also demonstrated a relationship between hand/finger movement and language ability (Nishimura and Matsuno, 1978); however, the evaluation criteria for Japanese language in the present study showed no relationship between dexterity and linguistic skills in Japanese language classes. This may be because movement of the hands and fingers entails fine motor skills, which is not the same as dexterity in general, and that the language ability measured was different from the skills measured in the present study.

These differences between the results of previous studies of music and Japanese language classes indicate that “dexterity” itself is comprised of various elements.

In addition, this study did not show any relationship between second grade mathematics learning and repeated side jump and softball throwing tasks with respect to skill learning. Although the results of this study differ from those of some previous studies, “mathematical ability” in this study is defined as the ability to draw figures correctly using tools, rather than in terms of mathematical concepts.

The results of the relationship between mathematics and dexterity also show that “dexterity” itself is composed of various elements. Nonetheless, this research was unable to clarify the relationship between the development of abstract concepts and the development of fine physical movement.

However, the findings of this study show that there may be a relationship between the repeated side jump and the skill elements required in arts and crafts up through the fourth grade of elementary school. The relationship between the softball throw, which has more skill points than the repeated side jump, and the skill elements required in arts and crafts was seen until about the third grade but not in the fourth grade. In other words, fine movement and gross movement were not differentiated until the third to fourth grades of elementary school but might be differentiated after that. In our recent study, the results of the 2016 physical fitness test before the COVID-19 pandemic and the results of the 2022 physical fitness test showed that at the elementary school where this study was conducted, there was a significantly lower difference in records in softball throwing and repeated side jump for fourth grade boys. This may be because the children did not have enough opportunities to gain sufficient exercise experience over the last two to three years. Being deprived of the opportunity to skillfully move their body may have consequences in the future.

In Japanese elementary schools, an individual homeroom teacher is essentially responsible for teaching all subjects. Teachers can promote learning in multiple subjects rather than just one by providing instruction that is tailored to the individual, with an awareness of physical developmental factors such as the dexterity of individual children. This, in turn, may promote self-confidence (i.e., increase self-efficacy) in children, which can have a positive effect on learning in later life. This demonstrates one of the possibilities of early education. Children having the idea that you can do something at an early age can have a positive effect on their future growth. Thus, there is a great significance to learning-related activities that can improve children’s dexterity.

### Limitations of the study and future lines of research

The limitations of this study and the issues to be addressed in the future are as follows. Further detailed investigation of differentiation and integration of motor skills in childhood should be conducted (e.g., create an observational/experimental design that considers the effects of growth and development, as well as ethics). The evaluation criteria included many aspects dependent on the cognitive and emotional elements that each subject emphasizes. As such, the functions measured by the repeated side jump and softball throw tasks may be different, meaning that it is not possible to make a general comparison. Therefore, in future studies it is important to make sure that the conditions are comparable. There is a need to clarify the differentiation and integration of motor skills by increasing the population parameter (i.e., number of participants) to further improve the reliability of the study and by conducting a follow-up survey. Additionally, the population parameters should also be expanded by excluding emotional factors from the evaluation criteria of subject learning, ensuring that evaluations are conducted as objectively as possible, and including a greater number of teachers in the research. Further, future research should explore the relationship between learning transfer and dexterity.

In recent years, research related to exercise has focused on the cerebellum. Research on fine and coarse motion needs to focus on using f-MRI in the future.

## Conclusion

Among students in the second to fourth grades of elementary school, this study found a significant difference between the group with a higher level of performance and the one with a middle or lower level of performance in terms of the number of repeated side jumps that participants were capable of (physical developmental factor) and their grades in terms of skills (subject learning) in the subject of arts and crafts.

Engaging in physical activity from early childhood may serve to prepare children for learning in the various subjects that they will encounter at school. Furthermore, the physical confidence derived from such activities can also serve as a foundation for participating in sports through the course of their life.

Given that elementary school teachers spend significant amounts of time with children every day teaching subjects including physical education and music, it may be meaningful for them to be aware of dexterity as being of significance across multiple subjects and to provide school education from the viewpoint of promoting and supporting development, regardless of how it is framed as a subject. It is meaningful to be able to teach in the right manner at the right time, thereby effectively improving the child’s abilities and enhancing self-esteem by increasing dexterity, especially during early childhood. This allows for an approach that engages children in physical activities from early childhood, which can also help in preparing them for learning a variety of subjects in school.

## Data availability statement

The original contributions presented in this study are included in the article/supplementary material, further inquiries can be directed to the corresponding author.

## Ethics statement

The studies involving human participants were reviewed and approved by this study was based on Ethics No. (HM-29) of the organization to which I belong. Written informed consent from the participants’ legal guardian/next of kin was not required to participate in this study in accordance with the national legislation and the institutional requirements.

## Author contributions

The author confirms being the sole contributor of this work and has approved it for publication.
